# Thyrotroph Hyperplasia Caused by Severe Primary Hypothyroidism Leading to Adrenal Crisis

**DOI:** 10.1210/jcemcr/luae187

**Published:** 2024-10-16

**Authors:** Yasser Hakami, Abdulaziz AlJaman

**Affiliations:** Obesity, Endocrine and Metabolism Center, King Fahad Medical City, Riyadh 12231, Saudi Arabia; Endocrine Department, King Saud Medical Complex, Riyadh 12746, Saudi Arabia

**Keywords:** hypothyroidism, pituitary hyperplasia, pituitary apoplexy, hypopituitarism, adrenal crisis

## Abstract

Thyrotroph hyperplasia is commonly present but remains largely undiagnosed in primary hypothyroidism. It is easily reversible with thyroid replacement therapy. If imaging is performed prior to biochemical evaluation, then patients may undergo pituitary surgery unnecessarily. We present the case of a 30-year-old man with thyrotroph hyperplasia caused by profound primary hypothyroidism leading to hypopituitarism that resolved after levothyroxine replacement therapy. We will discuss the current literature regarding pituitary hyperplasia in primary hypothyroidism in adults.

## Introduction

Thyrotroph hyperplasia refers to an absolute increase in the number of thyrotroph cells, resulting radiologically as a diffuse and symmetrical pituitary enlargement (pituitary hyperplasia) beyond normal parameters. Pituitary hyperplasia is a relatively common condition that occurs in physiological conditions, such as pregnancy and lactation (1), as well as pathological conditions, such as end-organ insufficiency conditions, including primary gonadal insufficiency, primary adrenal insufficiency, and primary hypothyroidism, owing to the loss of negative feedback (2). Primary hypothyroidism is responsible for approximately 33.3% of pituitary hyperplasia caused by end-organ insufficiency (3). The frequency of pituitary hyperplasia ranges from 25% to 81% in primary hypothyroidism (4). Prolonged primary hypothyroidism causes pituitary hyperplasia owing to the loss of negative feedback resulting from a deficiency of circulating thyroxine (T4) and triiodothyronine (T3). Additionally, excessive secretion of thyrotropin-releasing hormone from the hypothalamus leads to increased prolactin secretion. Furthermore, pituitary hyperplasia can reduce the release of other pituitary hormones, causing hypopituitarism, which may result in central adrenal insufficiency.

Despite the observed pituitary enlargement, thyrotroph cells comprise only 5% to 10% of adenohypophyseal cells. In patients with hypothyroidism, the number of thyrotroph cells can increase significantly to 34% of all adenohypophyseal cells (4). Pituitary hyperplasia can progress rapidly within a few weeks after inducing a hypothyroidism state intentionally. This scenario can occur during the withdrawal of levothyroxine replacement for preparing radioactive iodine therapy in patients with differentiated thyroid carcinoma. However, in these cases, the pituitary size can decrease after 5 weeks of resuming thyroid hormone replacement (5). Here, we report a case of a 30-year-old man who presented with features of adrenal crisis secondary to pituitary enlargement.

## Case Presentation

A 30-year-old man presented with a 3-day history of abdominal pain, nausea, recurrent vomiting, and blurred vision. He had been experiencing worsening headaches that lasted a month, as well as generalized weakness, easy fatigability, and constipation over the last several months. He had no galactorrhea, substantial weight change, or other symptoms of hypothyroidism. The patient denied receiving iodine or radiation therapy and had never undergone a computed tomography (CT) scan with contrast. He was not on any medications that could cause hypothyroidism or affect thyroid function. Additionally, he reported no upper respiratory tract infections within the last year. The patient had no family history of thyroid disorders. On physical examination, the patient was dehydrated and drowsy, with dry skin and mucous membranes. He was not pale or jaundiced and had no hyperpigmentation. His temperature was 36.8 °C. His blood pressure was 90/65 mm Hg. His pulse was 110 beat per minute. His O_2_ saturation was 95% on room air, and his respiratory rate was 22 breaths per minute. Eye examination revealed intact visual acuity, visual field, and extraocular movements. The patient had a goiter with an estimated size of 40 g in weight. Abdomen, cardiovascular system, chest, and central nervous system examinations were unremarkable, apart from a delay in the relaxation phase of his deep tendon reflexes.

## Diagnostic Assessment

**Figure 1.**
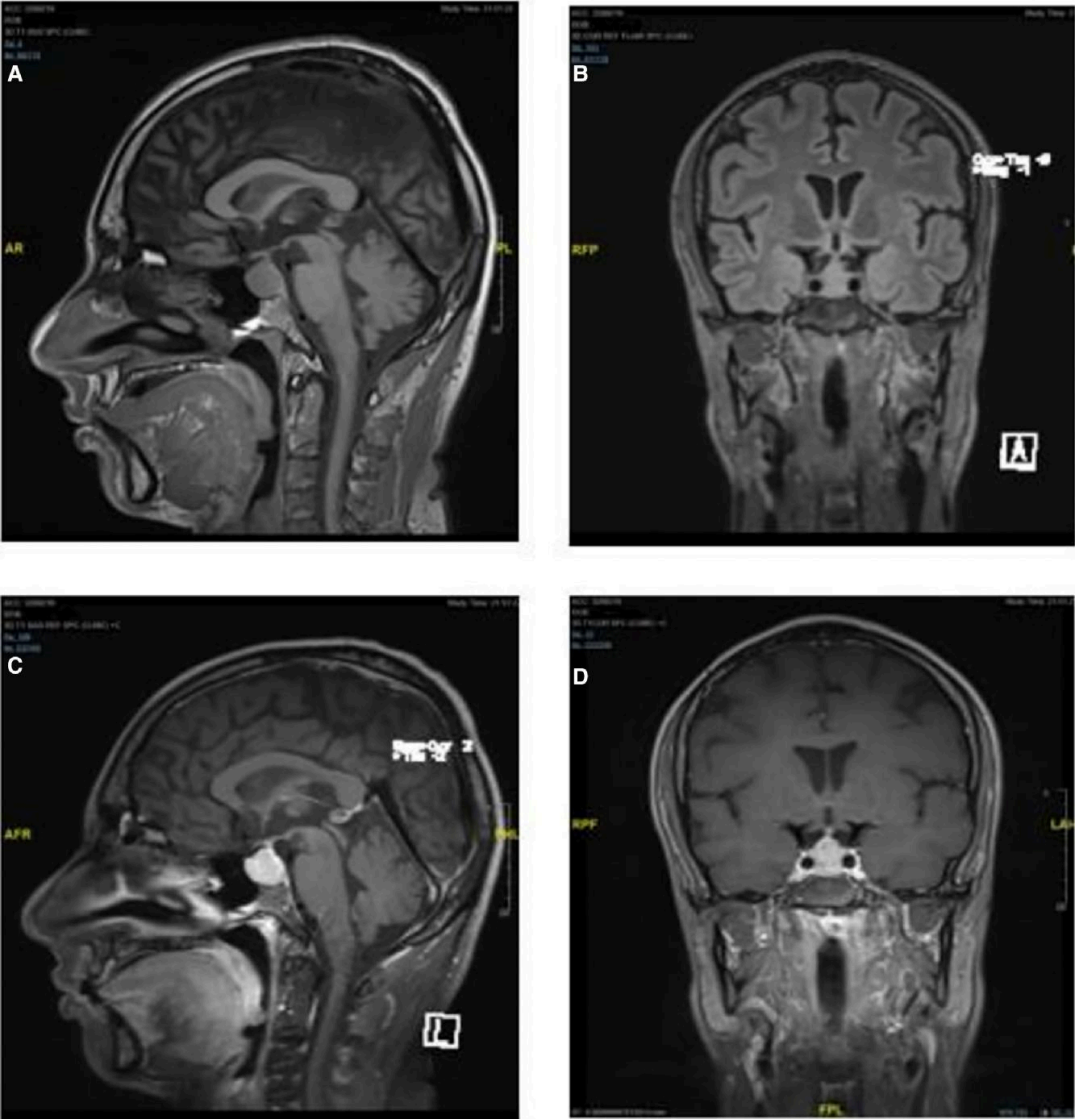
Initial pituitary magnetic resonance imaging scan displaying diffuse pituitary gland enlargement. (A) and (B) Unenhanced sagittal and coronal images indicated an enlarged pituitary gland with no compression on the optic chiasm. (C) and (D) Homogeneously enhanced sagittal and coronal images of the enlarged pituitary gland after Gd-DTPA.

Basic laboratory tests revealed a normal complete blood count, liver function tests, and serum creatine levels. His serum levels of urea, Na, and, K were 8.2 mmol/L (1.8-7.1 mmol/L), 132 mmol/L (136-142 mmol/L), and 3.6 mmol/L (3.5-5 mmol/L), respectively. Brain CT scan without contrast revealed a heterogeneously hyperdense sellar mass with mild suprasellar extension measuring 1.9 × 1.5 × 1.6 cm in transverse, anteroposterior, and craniocaudal dimensions, respectively. The patient was diagnosed with adrenal crisis secondary to possible pituitary apoplexy and was admitted for medical management and close observation. The pituitary hormonal panel revealed the following results: thyrotropin (TSH) > 1000 mIU/L (0.5-5.0 mIU/L) (SI: >1000 IU/L [0.5-5.0 IU/L]), free T4: 0.39 ng/dL (0.8-1.8 ng/dL) (SI: 5.1 pmol/L [10.30-23.17 pmol/L]), free T3: 1.10 pg/mL (2.3-4.2 pg/mL) (SI: 1.7 pmol/L [3.53-6.45 pmol/L]), prolactin: 117 ng/mL (4-23 ng/mL) (SI: 4.97 nmol/L [0.17-1.00 nmol/L]), cortisol: 1.92 µg/dL (5-25 µg/dL) (SI: 53 nmol/L [137.9-689.7 nmol/L]), adrenocorticotropin (ACTH): 12.72 pg/mL (10-60 pg/mL) (SI: 2.8 pmol/L [2.2-13.2 pmol/L]), total testosterone: 119.1 ng/dL (300-900 ng/dL) (SI: 4.13 nmol/L [10.4-31.2 nmol/L]), follicle-stimulating hormone (FSH): 9.2 mIU/mL (1.0-13.0 mIU/mL) (SI: 9.2 IU/L [1.0-13.0 IU/L]), and luteinizing hormone (LH): 1.2 mIU/mL (1.0-9.0 mIU/mL) (SI: 1.2 IU/L [1.0-9.0 IU/L]) (Table 1). His thyroperoxidase antibody level was 3.14 IU/mL (0.0-2.00 IU/mL) (SI: 3.14 kIU/L) [0.0-2.00 kIU/L]), and his thyroglobulin antibody level was 6.39 IU/mL (0.0-4.00 IU/mL) (SI: 6.39 kIU/L [0.0-4.00 kIU/L]). Brain magnetic resonance image (MRI) with contrast revealed diffuse enlargement of the anterior pituitary with increased upward convexity. However, no intrapituitary nonenhancing lesions were observed, suggesting the presence of pituitary adenoma. Mild thickening of the pituitary stalk, which remained centrally located, was noted. The optic chiasm remained intact. The posterior pituitary gland showed preserved spontaneous hyperintensity. The posterior fossa structures were grossly unremarkable (Fig. 1). These findings suggested the possibility of lymphocytic hypophysitis or pituitary hyperplasia rather than pituitary apoplexy.

**Table 1.** Plasma hormone concentrations over time

Hormone tested	Baseline	After 3 mo	After 6 mo	After 12 mo	Normal range
Cortisol	1.92 µg/dL (53 nmol/L)	10.40 µg/dL (287 nmol/L)	4.96 µg/dL (137 nmol/L)	14.06 µg/dL (388 nmol/L)	2-25 µg/dL (137.9-689.7 nmol/L)
ACTH	12.7 pg/mL (2.8 pmol/L)	20.4 pg/mL (4.5 pmol/L)	15.9 pg/mL (3.5 pmol/L)	5.9 pg/mL (1.3 pmol/L)	10-60 pg/mL (2.2-13.2 pmol/L)
LH	1.2 mIU/mL (1.2 IU/L)	3.3 mIU/mL (3.3 IU/L)	2.6 mIU/mL (2.6 IU/L)	3.1 mIU/mL (3.1 IU/L)	1-9 mIU/mL (1-9 IU/L)
FSH	9.2 mIU/mL (9.2 IU/L)	2.6 mIU/mL (2.6 IU/L)	3.1 mIU/mL (3.1 IU/L)	2.8 mIU/mL (2.8 IU/L)	1-13 mIU/mL (1-13 IU/L)
Total testosterone	119 ng/dL (4.13 nmol/L)	718.1 ng/dL (24.9 nmol/L)	643.1 ng/dL (22.3 nmol/L)	545 ng/dL (18.9 nmol/L)	300-900 ng/dL (10.4-31.2 nmol/L)
Prolactin	117 ng/mL (4.97 nmol/L)	139 ng/mL (5.9 nmol/L)	73.6 ng/mL (3.12 nmol/L)	37.8 ng/mL (1.6 nmol/L)	4-23 ng/mL (0.17-1.0 nmol/L)
TSH	>1000 mIU/L (>1000 IU/L)	37.9 mIU/L (37.9 IU/L)	8.2 mIU/L (8.2 IU/L)	0.6 mIU/L (0.6 IU/L)	0.5-5.0 mIU/L (0.5-5.0 IU/L)
Free T4	0.39 ng/dL (5.1 pmol/L)	0.92 ng/dL (11.9 pmol/L)	1.08 ng/dL (14 pmol/L)	1.21 ng/dL (15.7 pmol/L)	0.8-1.8 ng/dL (10.30-23.17 pmol/L)

Values in parenthesis are International System of Units (SI).

Abbreviations: ACTH, adrenocorticotropin; FSH, follicle-stimulating hormone; LH, luteinizing hormone; T4, thyroxine; TSH, thyrotropin.

## Treatment

During admission, the patient received intravenous fluid and was started on stress-dose hydrocortisone, followed by levothyroxine replacement therapy. Over the next few days, his clinical status improved significantly, and his urea and electrolyte levels normalized. He was discharged on hydrocortisone (10 mg morning and 5 mg evening) and levothyroxine (75 µg daily), with frequent follow-up visits to adjust the doses according to his hormonal profile.

## Outcome and Follow-up

**Figure 2.**
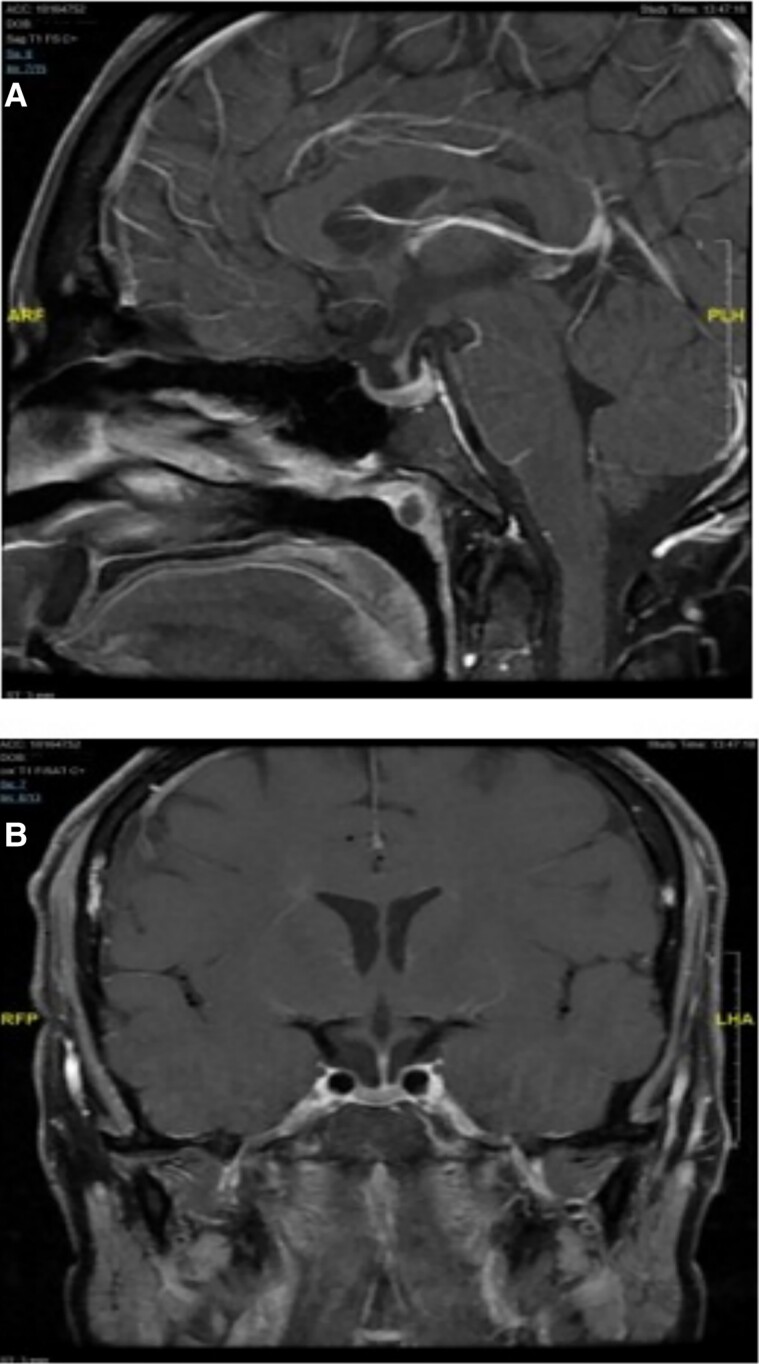
Gd-DTPA–enhanced pituitary magnetic resonance imaging scan 6 months after initiating thyroid hormone replacement therapy. (A) and (B) Sagittal and coronal images displaying a significant reduction in pituitary size.

Subsequently, the patient remained asymptomatic with normal blood pressure and unremarkable physical examination. The pituitary hormonal profile gradually improved (see Table 1). A follow-up MRI scan revealed a significant reduction in pituitary gland size (Fig. 2), suggesting that it was less likely to be a case of lymphocytic hypophysitis.

The latest laboratory tests indicated serum morning cortisol level of 14.06 µg/dL (SI: 338 nmol/L), TSH level of 0.60 mIU/L (SI: 0.60 IU/L), free T4 level of 1.21 ng/dL (SI: 15.7 pmol/L), and prolactin level of 37.8 ng/mL (SI: 1.60 nmol/L). The latest pituitary MRI scan revealed normal pituitary gland size with no signs of macroadenoma or abnormal intracranial enhancement. Hydrocortisone was tapered off successfully, whereas levothyroxine was maintained as a lifelong replacement therapy. Our final diagnosis was thyrotroph hyperplasia secondary to severe primary hypothyroidism, which led to hypopituitarism, and subsequently resolved with levothyroxine replacement therapy. The cause of the primary hypothyroidism is Hashimoto thyroiditis.

## Discussion

The first case of pituitary hyperplasia secondary to severe hypothyroidism was diagnosed by autopsy in 1851 (6) and then was described in a myxedema state by Boyce and Beadles in 1892 (7). Since then, several cases of sellar enlargement and chronic, long-standing hypothyroidism have been reported (8).

Scheithauer et al (9) conducted autopsies on patients with primary hypothyroidism and revealed the presence of diffuse thyrotropic cell hyperplasia in 69% and nodular thyrotropic cell hyperplasia in 25% of pituitary glands. A direct correlation was observed between the degree of thyrotropic cell hyperplasia and the absence of negative feedback on thyrotropic cells following thyroid hormone replacement therapy. In 1976, Yamada et al (10) suggested that 81% of patients with hypothyroidism had an abnormal increase in the volume of the sella turcica, and the volume was directly related to the level of TSH. Khawaja et al (11) reported that up to 70% of patients with TSH levels of 50 mIU/L or greater had pituitary enlargement. The size of the enlarged pituitary gland was significantly correlated with the TSH levels.

Primary hypothyroidism has a chronic course with insidious and gradual onset of signs and symptoms, for which assessment and management are occasionally delayed. Iodine deficiency is the most common cause of primary hypothyroidism worldwide, while the most common cause of primary hypothyroidism in iodine-sufficient areas is chronic autoimmune (Hashimoto) thyroiditis. Thyroidectomy, external radiation therapy, and radioiodine treatment are well-known examples of iatrogenic causes of hypothyroidism. Furthermore, some drugs used to treat nonthyroidal conditions can cause hypothyroidism. These drugs include amiodarone, lithium carbonate, interferon α, checkpoint inhibitor immunotherapy, and tyrosine kinase inhibitors. Symptoms and signs of primary hypothyroidism vary in relation to the magnitude of thyroid hormone deficiency. At initial presentation, only 38% of patients present with symptoms of hypothyroidism, 25% present with compressive signs of pituitary hyperplasia, and 36% present with features of hyperprolactinemia (4). Honbo et al (12) reported in 1978 that serum prolactin levels were elevated in 39% of patients with untreated primary hypothyroidism. The direct correlation between serum TSH and prolactin levels suggests that hyperprolactinemia in primary hypothyroidism is primarily mediated by thyrotropin-releasing hormone release in the absence of negative feedback from T3 and T4. Thyrotroph hyperplasia may cause chiasmal compression (13). Long-standing pituitary hyperplasia may result in pituitary injury, leading to partial or complete hypopituitarism, which can be permanent (14). Our patient presented with all these major clinical components of primary hypothyroidism. Furthermore, he developed partial hypopituitarism, which was resolved with levothyroxine replacement therapy.

The differential diagnosis of pituitary hyperplasia includes functioning and nonfunctioning pituitary adenomas. The diagnosis of thyrotroph pituitary hyperplasia initially depends on thyroid function tests and imaging studies. In patients with high TSH levels and MRI evidence of pituitary enlargement, it is important to differentiate between thyrotroph (TSH-producing) adenoma and thyrotroph hyperplasia to avoid inappropriate pituitary surgery. However, TSH-producing pituitary adenomas present with high or inappropriately normal TSH levels associated with elevated thyroid hormone levels.

Imaging techniques such as CT and MRI currently have low sensitivity for distinguishing between pituitary hyperplasia and macroadenoma. MRI is superior to CT for imaging the pituitary gland and for further follow-ups to monitor changes in pituitary size (15). The MRI characteristics of pituitary macroadenoma include homogeneous enlargement of the gland with a height greater than 1 cm, which can be associated with perisellar extension and pituitary stalk deviation. These findings can also be observed in pituitary hyperplasia (16). Therefore, imaging studies are insufficient to reach a definitive diagnosis. However, biochemical and radiological responses to levothyroxine favor hyperplasia over adenoma. Regression of pituitary enlargement with levothyroxine replacement therapy can confirm the diagnosis (17).

The time of clinical response is variable. In some cases of hypothyroidism, pituitary size can decrease after 5 weeks of thyroid hormone replacement (5). In another trial of thyroxine replacement therapy for 6 to 12 weeks, an MRI after 12 weeks helped to diagnose pituitary hyperplasia correctly (18). Most radiological responses varied from a few weeks to several months. Thus, appropriate diagnosis and treatment are extremely important, as they can help to avoid unnecessary surgery or treatment with dopamine agonist agents.

To confirm the diagnosis pathologically, surgery can be considered if there is no response to levothyroxine replacement therapy in terms of pituitary size. Unrecovered failure could also lead to the consideration of surgery. Furthermore, surgical management can be considered as a means of decompression if the optic chiasm is affected and vision is compromised (13). Our patient responded well to levothyroxine replacement therapy. His follow-up MRI scan revealed a regression in pituitary enlargement (see Fig. 2).

Our patient presented with a unique case of acute adrenal crisis as the main clinical finding. This acute presentation usually occurs in patients with primary or secondary adrenal insufficiency who had a major physical or psychologically stressful event or if the patient is noncompliant with hydrocortisone replacement therapy (19). In many cases, adrenal crisis can be the initial presentation of a newly diagnosed primary adrenal insufficiency (eg, Addison disease) or secondary adrenal insufficiency (eg, hypopituitarism) (20).

Adrenal crisis secondary to thyrotroph hyperplasia caused by severe primary hypothyroidism is a highly rare clinical presentation. Furthermore, the literature review about this is very limited. Hence, it is crucial to differentiate between pituitary hyperplasia and pituitary apoplexy in patients with acute adrenal crisis and pituitary enlargement. With a proper diagnosis, unnecessary surgical intervention can be avoided.

Treating such conditions promptly and appropriately is critical because delayed treatment can increase the mortality rate. Our patient received proper management without delay, which resulted in considerable improvement of his general condition, with complete resolution of hypogonadism and adrenal insufficiency. Only levothyroxine therapy was required for our patient to prevent the recurrence of his condition.

In conclusion, hypopituitarism secondary to thyrotroph hyperplasia caused by severe primary hypothyroidism is rare, and the literature review about this clinical condition is scarce. Our findings suggest that thyrotroph hyperplasia secondary to primary hypothyroidism should be considered as a differential diagnosis of diffuse pituitary enlargement with high TSH levels. An experienced multidisciplinary team must evaluate such scenarios to appropriately assess and manage the case. Appropriate medical management and careful monitoring are the mainstays of adequate treatment, which helps to avoid unnecessary surgery and dopamine agonist therapy. In contrast, levothyroxine therapy results in the resolution of the pituitary enlargement, which leads to complete recovery of pituitary function in most cases. Thus, knowledge of this entity is of paramount importance.

## Learning Points

- Pituitary hyperplasia is defined as an absolute increase in the number of pituitary trophic cells. It is a relatively common condition that occurs in physiological as well as pathological situations.
- Pathological hyperplasia occurs in end-organ insufficiency conditions such as primary gonadal insufficiency, primary adrenal insufficiency, and primary hypothyroidism, due to the loss of negative feedback.
- Long-standing pituitary hyperplasia may result in pituitary injury leading to partial or complete hypopituitarism, which can be permanent.
- The diagnosis of thyrotroph pituitary hyperplasia initially depends on thyroid function test and imaging studies. Biochemical and radiological responses to levothyroxine favor hyperplasia over adenoma. Thus, regression of pituitary enlargement with levothyroxine replacement therapy can confirm the diagnosis.

## Contributors

Both authors made individual contributions to authorship. Y.H. and A.A. were involved in the diagnosis and management of this patient and manuscript preparation and submission. Both authors reviewed and approved the final draft.

## Data Availability

Some data sets generated during and/or analyzed during this study are not publicly available but are available from the corresponding author on reasonable request.

## Abbreviations

ACTH: adrenocorticotropin
CT: computed tomography
FSH: follicle-stimulating hormone
LH: luteinizing hormone
MRI: magnetic resonance imaging
T3: triiodothyronine
T4: thyroxine
TSH: thyrotropin
